# Neuronal deletion of the circadian clock gene *Bmal1* induces cell-autonomous dopaminergic neurodegeneration

**DOI:** 10.1172/jci.insight.162771

**Published:** 2024-01-23

**Authors:** Michael K. Kanan, Patrick W. Sheehan, Jessica N. Haines, Pedro G. Gomez, Adya Dhuler, Collin J. Nadarajah, Zachary M. Wargel, Brittany M. Freeberg, Hemanth R. Nelvagal, Mariko Izumo, Joseph S. Takahashi, Jonathan D. Cooper, Albert A. Davis, Erik S. Musiek

**Affiliations:** 1 Department of Neurology and; 2 Departments of Pediatrics, Genetics and Genomic Medicine, Washington University School of Medicine, St. Louis, Missouri, USA.; 3 Department of Neuroscience and; 4 Howard Hughes Medical Institute, University of Texas Southwestern Medical Center, Dallas, Texas, USA.; 5 Center On Biological Rhythms and Sleep (COBRAS), Washington University School of Medicine, St. Louis, Missouri, USA.

**Keywords:** Aging, Neuroscience, Neurodegeneration, Parkinson disease

## Abstract

Circadian rhythm dysfunction is a hallmark of Parkinson disease (PD), and diminished expression of the core clock gene *Bmal1* has been described in patients with PD. BMAL1 is required for core circadian clock function but also serves nonrhythmic functions. Germline *Bmal1* deletion can cause brain oxidative stress and synapse loss in mice, and it can exacerbate dopaminergic neurodegeneration in response to the toxin MPTP. Here we examined the effect of cell type–specific *Bmal1* deletion on dopaminergic neuron viability in vivo. We observed that global, postnatal deletion of *Bmal1* caused spontaneous loss of tyrosine hydroxylase^+^ (TH^+^) dopaminergic neurons in the substantia nigra pars compacta (SNpc). This was not replicated by light-induced disruption of behavioral circadian rhythms and was not induced by astrocyte- or microglia-specific *Bmal1* deletion. However, either pan-neuronal or TH neuron–specific *Bmal1* deletion caused cell-autonomous loss of TH^+^ neurons in the SNpc. *Bmal1* deletion did not change the percentage of TH neuron loss after α-synuclein fibril injection, though *Bmal1*-KO mice had fewer TH neurons at baseline. Transcriptomics analysis revealed dysregulation of pathways involved in oxidative phosphorylation and Parkinson disease. These findings demonstrate a cell-autonomous role for BMAL1 in regulating dopaminergic neuronal survival and may have important implications for neuroprotection in PD.

## Abstract 2

<p>The circadian clock protein BMAL1 regulates survival of dopaminergic neurons in vivo in a cell-autonomous manner.</p>

## Introduction

Circadian clocks regulate many aspects of cellular function, including cellular metabolism, redox homeostasis, and inflammation (1, 2). On a molecular level, the core circadian clock consists of a transcriptional-translational feedback loop that tunes gene expression and cellular function to a 24-hour rhythm. This core circadian clock consists of the basic helix-loop-helix transcription factor BMAL1 (also referred to as ARNTL), which heterodimerizes with CLOCK or NPAS2 to drive tissue-specific transcriptional programs. BMAL1 drives expression of several negative-feedback repressors, including PER, CRY, and REV-ERB proteins, which in turn inhibit BMAL1-mediated transcription and are tuned through posttranscriptional regulation to a 24-hour period (3). BMAL1 is required for circadian oscillations in transcription, cellular function, and behavior, and *Bmal1* deletion renders mice arrhythmic at both cellular and behavioral levels (4). While binding of BMAL1 to DNA is highly rhythmic, BMAL1 also regulates expression of many nonrhythmic transcripts and is, thus, thought to exert additional “noncircadian” functions (5, 6). Our group has previously shown that *Bmal1* deletion causes astrogliosis, oxidative stress, and synapse loss in mouse cerebral cortex (6, 7). However, overt neurodegeneration was not observed in *Bmal1*-KO mice in vivo in regions such as the hippocampus, cortex, and striatum (7). Furthermore, *Bmal1* deletion appears to have protective effects in some models of neurological disease, such as tau and α-synuclein (αSyn) aggregation, stroke, and spinal cord injury (8–10). Thus, the role of *Bmal1* in brain health and neurodegeneration is not entirely understood.

Parkinson disease (PD) is a common neurodegenerative disease characterized clinically by progressive tremor, rigidity, bradykinesia, postural instability, autonomic dysfunction, and neuropsychiatric symptoms. Key pathological hallmarks of PD include degeneration of dopaminergic neurons in the substantia nigra pars compacta (SNpc) and intraneuronal accumulation of aggregated αSyn in Lewy bodies and Lewy neurites. A considerable amount of literature describes circadian disruption in PD, including fragmentation of daily behavioral rhythms and blunting of rhythms of *Bmal1* and other core circadian clock gene expression in peripheral blood mononuclear cells (PBMCs) from patients with PD (11–15). In mice, both light-induced circadian disruption (16) and germline *Bmal1* deletion have been shown to exacerbate dopaminergic neurodegeneration caused by the mitochondrial toxin 1-methyl-4-phenyl-1,2,3,6-tetrahydropyridine (MPTP) (17). While a previous study implicated microglial dysfunction as critical to this phenotype, germline *Bmal1*-KO mice exhibit a variety of developmental phenotypes that could contribute, and a comprehensive cell type–specific analysis has not been conducted in vivo (17). Here, we examine the effects of postnatal and cell type–specific *Bmal1* deletion on viability of dopaminergic neurons in the SNpc and test the effect of *Bmal1* deletion on αSyn pathology and neurodegeneration in a mouse model. Our data indicate that *Bmal1* regulates specific cell-autonomous transcriptional programs in dopaminergic neurons that are critical for their survival, suggesting that preserving *Bmal1* expression may be neuroprotective in PD.

## Results

**Figure 1:**
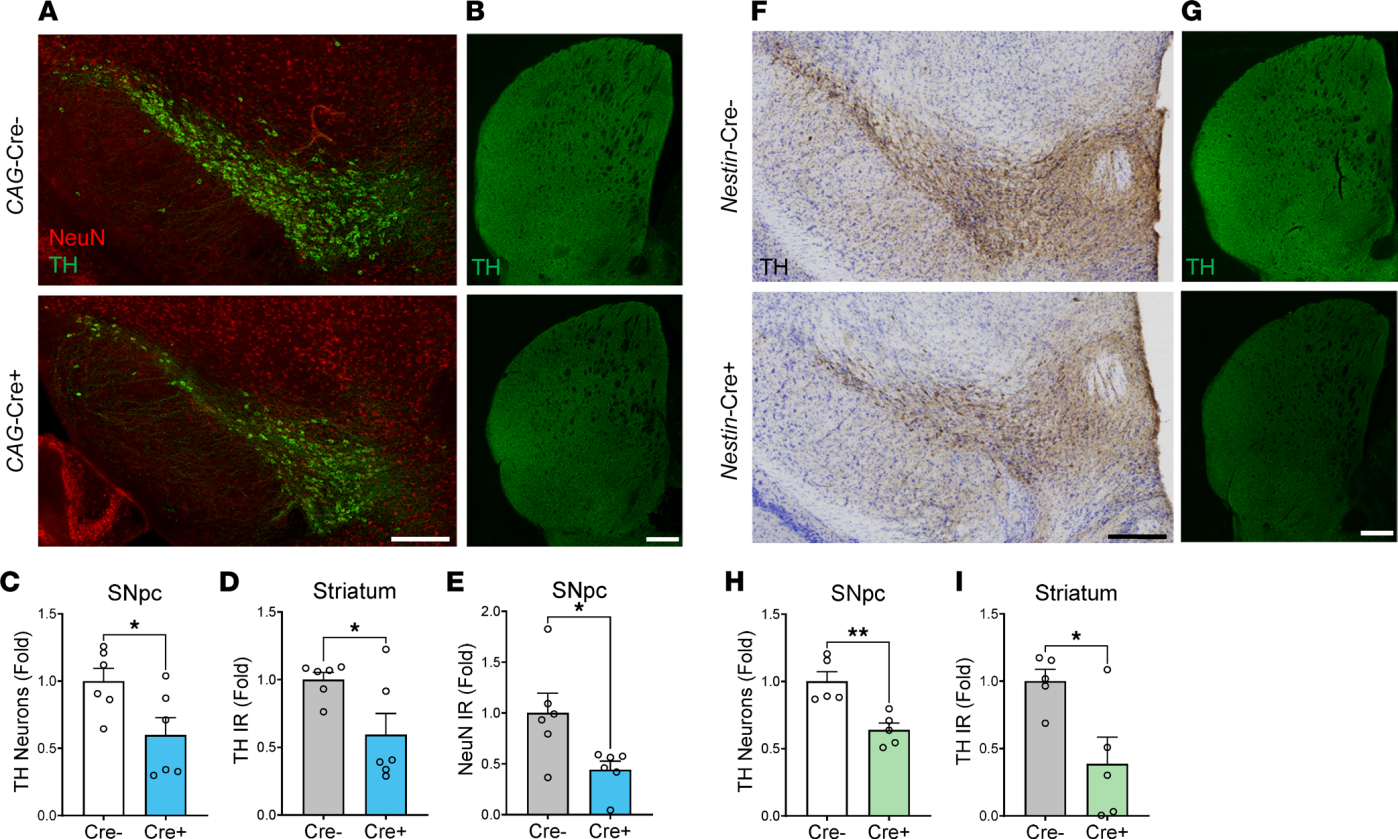
Global and brain-specific *Bmal1* deletion induces degeneration of dopaminergic neurons in the SNpc. (**A**) Representative images of TH (green) and NeuN (red) staining within the SNpc of Cre^–^;B*mal1*^fl/fl^ control (*CAG*-Cre^–^) and *CAG*-Cre^+^;*Bmal1*^fl/fl^ global *Bmal1*-KO mice (*CAG*-Cre^+^). Scale bar: 150 μm. (**B**) Representative images of TH staining in striatum of *CAG*-Cre^–^ and Cre^+^ mice. Scale bar: 200 μm. (**C**) Stereological counts of TH^+^ cells in the SNpc of *CAG*-Cre^–^ controls and *CAG*-Cre^+^ *Bmal1*-KO mice. *n* = 5–6 mice per genotype. Fold change normalized to average of Cre^–^ condition. (**D**) Quantification of striatal TH immunoreactivity (IR) intensity from images in **B**. Fold change normalized to average of Cre^–^ condition. (**E**) Quantification of NeuN IR (% area) in the SNpc of *CAG*-Cre^–^ controls and *CAG*-Cre^+^ *Bmal1*-KO mice. *n* = 6 mice per genotype. Fold change normalized to average of Cre^–^ condition. (**F**) Representative images of SNpc TH staining (brown) with H&E counterstaining (purple) in the SNpc of *Nestin*-Cre^–^;*Bmal1*^fl/fl^ control and *Nestin*-Cre^+^;*Bmal1*^fl/fl^ brain-specific *Bmal1*-KO mice. Scale bar: 150 μm. (**G**) Representative images of TH staining in striatum of *Nestin*-Cre^–^ and Cre^+^ mice. Scale bar: 200 μm. (**H**) Stereological counts of TH^+^ cells in the SNpc and *Nestin*-Cre^–^ control (Cre^–^) and *Nestin*-Cre^+^ brain-specific *Bmal1*-KO mice (Cre^+^). *n* = 5 mice per genotype. (**I**) Striatal TH IR intensity quantified from images in **G**. For **C**–**E**, **H**, and **I**, each circle represents averaged counts from a single mouse, and fold change was normalized to average of Cre^–^ condition. In all panels, data represent mean ± SEM. *P < 0.05, **P < 0.01 by 2-tailed *t* test.

We first examined the number of midbrain dopaminergic neurons in mice following tamoxifen-inducible, postnatal global *Bmal1* deletion (*CAG*-Cre^ERT2^;*Bmal1*^fl/fl^). We and others have previously characterized this mouse line and have shown that postnatal tamoxifen administration causes widespread *Bmal1* deletion in the brain as well as behavioral arrhythmicity, while avoiding multiple developmental complications noted in germline *Bmal1*-KO mice (18, 19). We performed unbiased stereology of tyrosine hydroxylase^+^ (TH^+^) neurons in the SNpc in Cre^–^ (control) and Cre^+^ (global *Bmal1*-KO) littermates, all treated with tamoxifen at 2–4 months old. One month after tamoxifen treatment, we observed a loss of BMAL1 immunoreactivity in the nuclei of TH^+^ neurons in the SNpc of Cre^+^ mice (Supplemental Figure 1A; supplemental material available online with this article; https://doi.org/10.1172/jci.insight.162771DS1). We observed a 40% decrease in the number of TH^+^ neurons in Cre^+^ mice compared with Cre^–^ mice (Figure 1, A–C). TH immunoreactivity in the striatum was also decreased by 41% in Cre^+^ mice compared with Cre^–^ mice (Figure 1, B and D). We also observed a reduction in number of NeuN^+^ neurons in the SNpc in *CAG*-Cre^+^ mice, and this was similar in magnitude as compared with the loss of TH^+^ cells, suggesting that neurodegeneration — rather than simply a reduction in TH protein expression — accounted for the apparent loss of TH^+^ neurons (Figure 1E). We also counted TH^+^ neurons in the ventral tegmental area (VTA), an adjacent brain region containing dopaminergic neurons but one that does not exhibit the same selective vulnerability as the SNpc in PD. TH^+^ neuron numbers were also reduced in the VTA (Supplemental Figure 1, B and C), suggesting a broad sensitivity of TH^+^ neurons to global deletion of *Bmal1*. However, NeuN^+^ neuronal nuclei were grossly intact in the hippocampus of Cre^+^ global KO mice compared with Cre^–^ mice, suggesting that hippocampal neurons do not degenerate in similar numbers as TH^+^ neurons, though detailed stereology was not performed (Supplemental Figure 1D). A previous study showed that REV-ERBα, a direct transcriptional target of BMAL1 that is suppressed in *Bmal1*-KO brain (7), suppresses *Th* gene expression in the midbrain (20). Thus, we would not expect that *Bmal1* deletion would directly suppress *Th* expression; rather, increased expression of *Th* gene might be expected. Accordingly, there was no change in *Th* mRNA levels in *Nestin*-Cre;*Bmal1*^fl/fl^ mouse cortex (Supplemental Figure 1E). Thus, it is unlikely that *Bmal1* deletion simply causes downregulation of the *Th* gene. Of note, neither C*AG*-Cre^ERT2+^;*Bmal1*^fl/fl^ mice, which were not treated with tamoxifen, nor tamoxifen-treated C*AG*-Cre^ERT2+^ mice, in which *Bmal1* is not floxed, showed any loss of TH^+^ neurons in the SNpc, demonstrating that Cre-related toxicity did not explain our findings (Supplemental Figure 2).

Global inducible *Bmal1*-KO mice are behaviorally arrhythmic under constant lighting conditions and have *Bmal1* deletion in all tissues (18, 19). In order to determine if disrupted behavioral rhythms or peripheral *Bmal1* deletion contributed to TH^+^ neuron loss, we examined the SNpc of 9- to 11-month-old *Nestin*-Cre^+^;*Bmal1*^fl/fl^ mice, which have deletion of *Bmal1* in neurons, astrocytes, and other cells throughout the brain but have intact *Bmal1* expression in peripheral organs as well as intact behavioral rhythms due to limited Cre-mediated deletion of *Bmal1* in the suprachiasmatic nucleus (SCN) (7, 18). *Nestin-Bmal1*–KO mice also exhibited a similar degree of loss of TH^+^ neurons in the SNpc and loss of TH staining in the striatum (Figure 1, F–I), demonstrating that degeneration is likely due to loss of *Bmal1* expression in neurons or astrocytes and not due to disrupted whole-animal behavioral circadian rhythms or peripheral organ dysfunction. Notably, *Nestin*-*Bmal1*–KO mice also retain microglial *Bmal1* expression, indicating that microglial *Bmal1* deletion is not required for the TH neurodegeneration phenotype.

**Figure 2:**
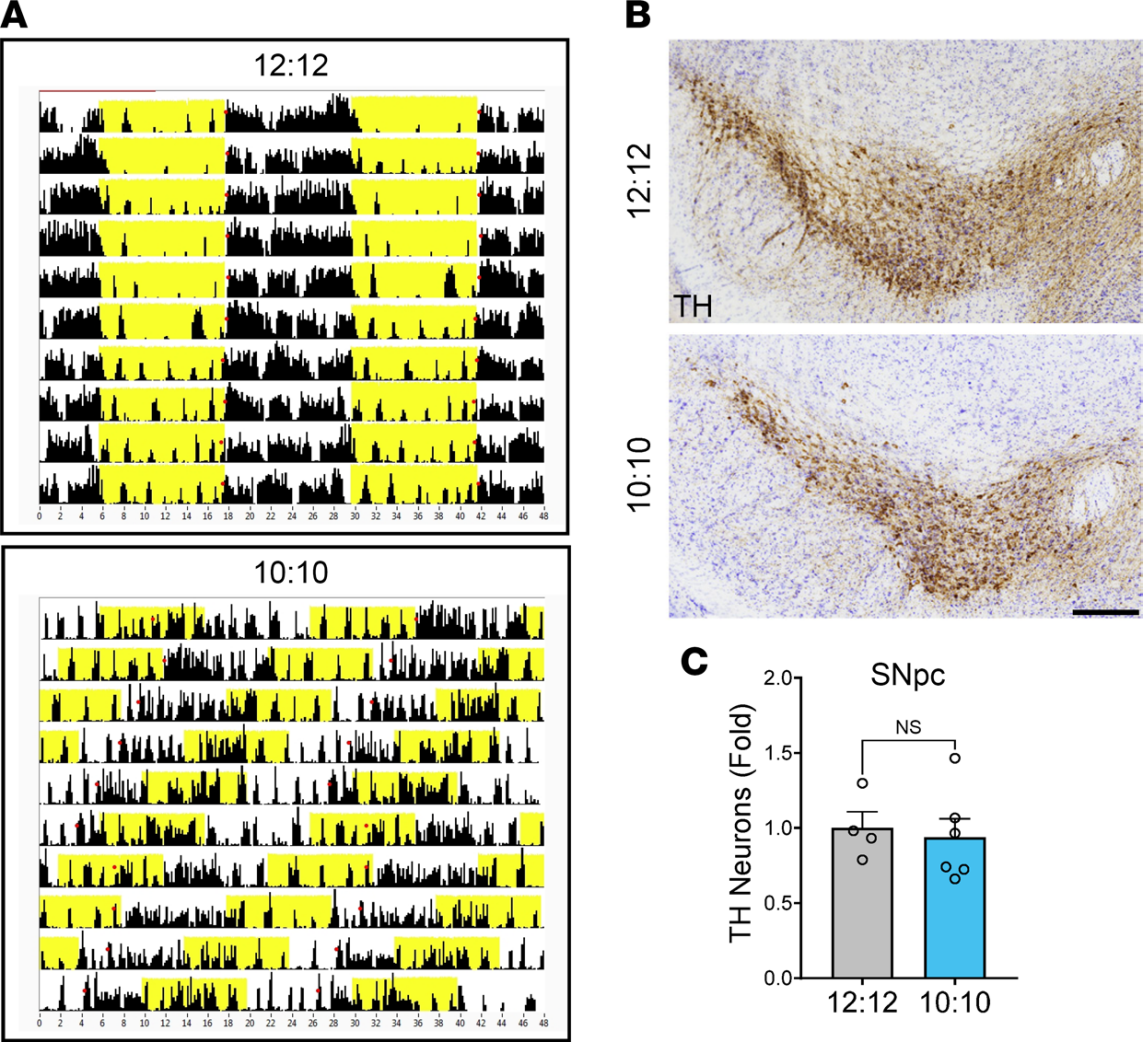
Light-induced circadian disruption does not induce TH^+^ neuron loss in the SNpc. (**A**) Representative actograms showing mouse activity (detected by infrared motion sensor) from mice subjected to control lighting (12-hour/12-hour [12:12] L:D) or 10:10 L:D circadian disruption paradigm. Double plotted; yellow indicates lights on. (**B**) Representative images of TH (brown) and cresyl violet (purple) staining in WT mice subjected to 10:10 or 12:12 lighting schedules from age 3 months old to 6 months old. Scale bar: 150 μm. (**C**) Stereological counts of TH^+^ cells within the SNpc of mice from **A**. *n* = 4–5 mice per group. ns, not significant by 2-tailed *t* test (*P* > 0.1). Each circle represents data from a single mouse. Fold change was normalized to the average of 12:12 condition. Data are shown as mean ± SEM.

In order to further investigate the role of whole animal circadian rhythm disruption in dopaminergic neurodegeneration, we subjected 3-month-old WT mice to circadian desynchrony via 12 weeks of exposure to a 10-hour/10-hour light/dark (L:D) cycle (T20 cycle) (21). Control mice were kept on standard 12-hour/12-hour L:D lighting. This intervention causes circadian desynchrony between the light cycle and the internal clock, and it has been shown to cause alterations in neuronal morphology and cognitive impairment in rodents (22). We observed disruption of behavioral circadian rhythms in mice subjected to the T20 cycle (Figure 2A), but this had no effect on TH^+^ neuron counts in the SNpc (Figure 2, B and C). This result suggests that disruption of behavioral circadian rhythms via altered lighting schedule does not phenocopy the effect of *Bmal1* deletion on TH neuron viability.

**Figure 3:**
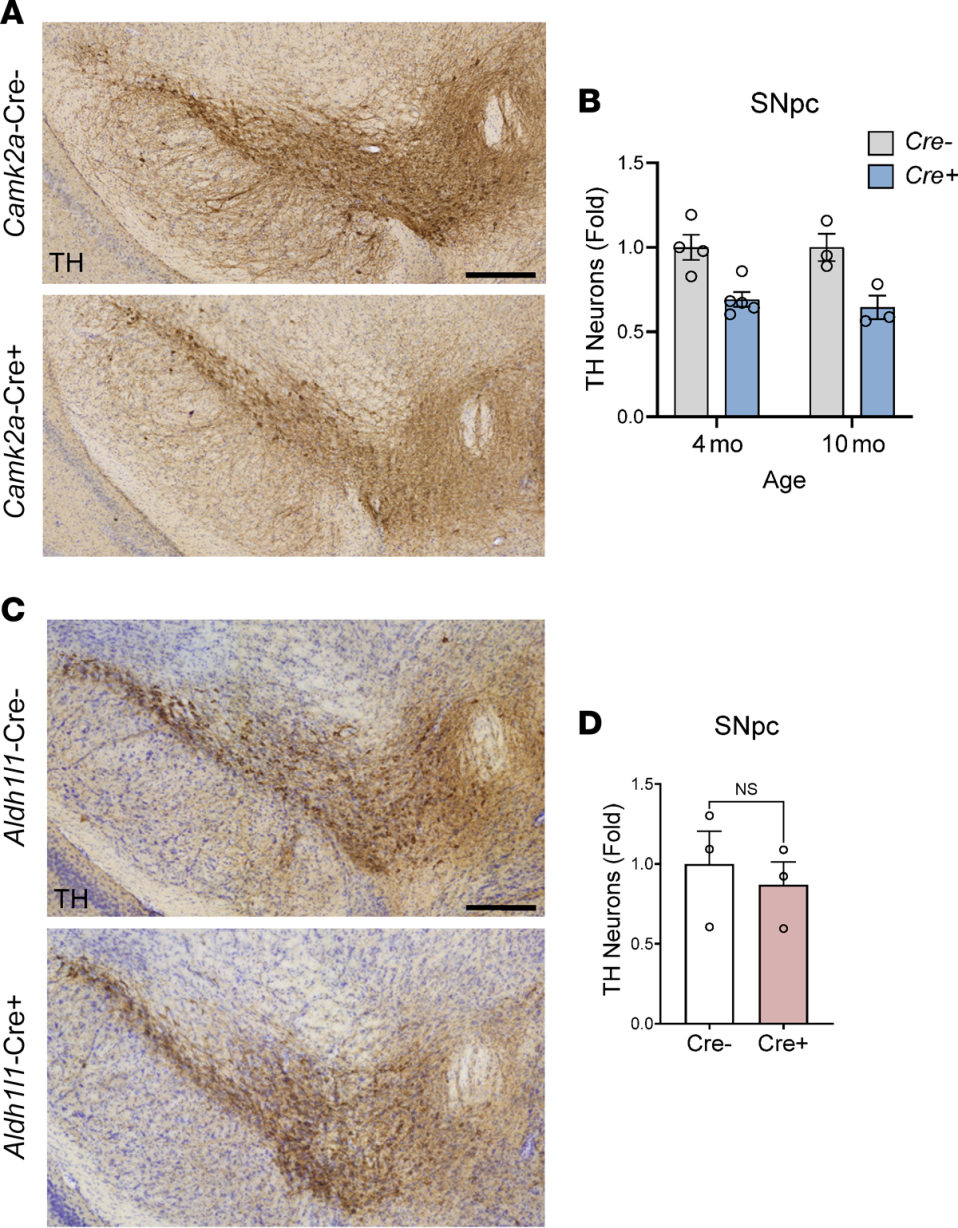
Deletion of *Bmal1* in neurons, but not astrocytes, induces dopaminergic neuron loss. (**A**) Representative images of TH (brown) and cresyl violet staining in the SNpc of Cre^–^;*Bmal*^fl/fl^ control and *Camk2a*-iCre^+^;*Bmal*^fl/fl^ neuron-specific *Bmal1*-KO mice. Scale bar: 150 μm. (**B**) Quantification of stereological counts of TH^+^ cells within the SNpc of 4- and 10-month cohorts of Cre^–^ control and Cre^+^ neuron-specific *Bmal1*-KO mice. *n* = 3–4 mice per group. Two-way ANOVA showed a significant main effect of Cre genotype (F[1,11] = 24.78, *P* = 0.0004) but no significant main effect of age or interaction (F[1,11] = 0.1217, *P* = 0.734). (**C**) Representative images of TH (brown) and cresyl violet staining in the SNpc of Cre^–^;B*mal*^fl/fl^ control and *Aldh1l1*-Cre^ERT2+^;*Bmal*^fl/fl^ astrocyte-specific *Bmal1*-KO mice, 2 months after tamoxifen. Scale bar: 150 μm. (**D**) Quantification of stereological counts of TH^+^ cells within the SNpc of Cre^–^ control and Cre^+^ astrocyte-specific *Bmal1*-KO mice. *n* = 3 mice per group. NS indicates *P* > 0.1 by 2-tailed *t* test. For **B** and **D**, each circle represents averaged counts from a single mouse, and fold change was normalized to average of Cre^–^ condition. In all panels, data represent mean ± SEM.

We next counted SNpc TH^+^ neurons in mice with neuron–specific *Bmal1* deletion. We used a particular line of BAC-transgenic *CaMK2a*-Cre;*Bmal1*^fl/fl^ mice, previously shown to have widespread, pan-neuronal *Bmal1* deletion and behavioral rhythm disturbances (6, 22). Both 4-month-old and 10-month-old *CaMK2a*-iCre;*Bmal1*^fl/fl^ mice exhibited a reduction in number of SNpc TH^+^ neurons as compared with Cre^–^ littermate controls (Figure 3, A and B), demonstrating a neuronal cell–autonomous effect of *Bmal1* deletion on TH^+^ cell survival. There was no main effect of age (4-month-old versus 10-month-old) on neuronal loss in *CaMK2a*-iCre;*Bmal1*^fl/fl^ mice and no interaction between genotype and age, suggesting that degeneration due to *Bmal1* deletion occurs early and then plateaus.

We have previously shown that mice with astrocyte-specific *Bmal1* deletion (*Aldh1l1*-Cre^ERT2^;*Bmal1*^fl/fl^) develop spontaneous astrogliosis and astroglial transcriptional alterations (6). To evaluate a role for astrocyte *Bmal1* in dopaminergic cell loss, we next examined TH^+^ neurons in the SNpc in *Aldh1l1*-Cre^ERT2^;*Bmal1*^fl/fl^ mice. One month after tamoxifen treatment, 4- to 5-month-old astrocyte *Bmal1*-KO mice showed no change in TH^+^ neuron counts in the SNpc compared with Cre^–^ littermate controls (Figure 3, C and D). Thus, *Bmal1* deletion in astrocytes does not account for the TH^+^ neuron loss phenotype observed in *Nestin-Bmal1*–KO and *CAG-Bmal1*–KO mice.

**Figure 4:**
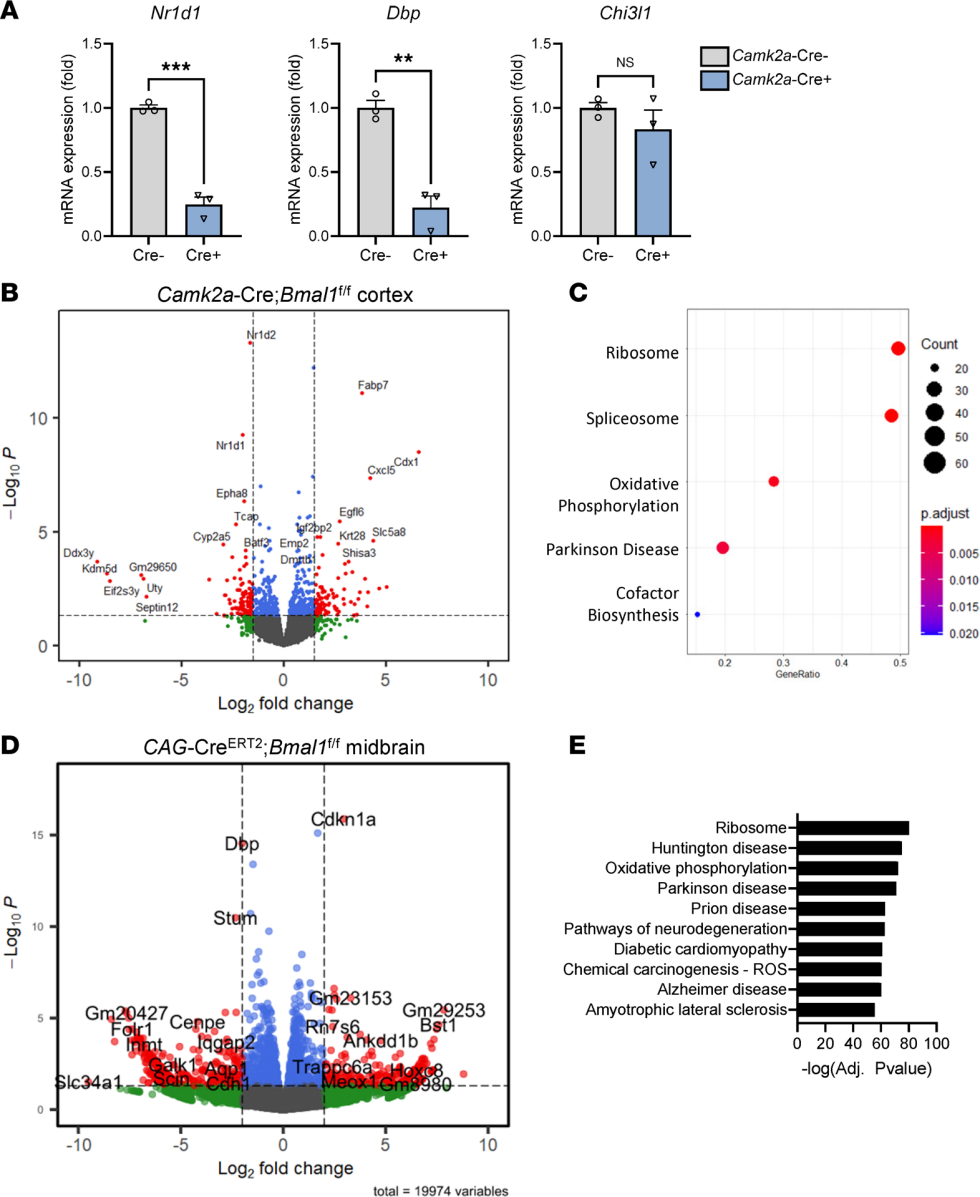
Transcriptomics analysis of midbrain tissue from *Bmal1*-KO mice reveals dysregulation of pathways related to Parkinson disease and oxidative phosphorylation. (**A**) Expression of known BMAL1 transcriptional targets in cortex from Cre^–^;*Bmal*^fl/fl^ control and *Camk2a*-iCre;*Bmal*^fl/fl^ neuron-specific *Bmal1*-KO mice at 4 months old. *n* = 3 mice/genotype. (**B**) Volcano plot showing differentially expressed genes (DEGs) in cortex tissue from *Camk2a*-iCre;*Bmal*^fl/fl^ neuron-specific *Bmal1*-KO mice, as compared with Cre^–^ controls. DEGs with fold change > 2 and uncorrected *P* < 0.05 are shown in red. Data are derived from *n* = 3 mice/genotype. (**C**) KEGG pathway analysis of all DEGs which were up- or downregulation in *Camk2a*-iCre;*Bmal*^fl/fl^ cortex with multiple-comparison adjusted *P* < 0.05. (**D**) Exploratory volcano plot showing DEGs (DEGs) in midbrain tissue from *CAG*-Cre^ERt2+^;*Bmal1*^fl/fl^ mice, as compared with Cre^–^ controls. DEGs with fold change > 2 and uncorrected *P* < 0.05 are shown in red. *Fabp7* is not shown but is highly significant and upregulated off scale. Data are derived from *n* = 3 Cre^–^ and *n* = 2 Cre^+^ mice. (**E**) Exploratory KEGG pathway analysis of all DEGs that were up- or downregulation in Cre^+^ midbrain samples with multiple-comparison adjusted *P* < 0.05.

In order to examine possible transcriptional mechanisms by which *Bmal1* deletion might influence neuronal survival, we performed bulk RNA-Seq on cerebral cortex tissue from 4-month-old pan-neuronal *Bmal1*-KO mice (*Camk2a*-*Bmal1*–KO [*Bmal1*^fl/fl^]) and Cre^–^ littermate controls (Figure 4A). The known BMAL1 transcriptional targets *Nr1d1* and *Dbp* were strongly downregulated in Cre^+^ mouse cortex, indicating loss of BMAL1 activity (Figure 4A). Utilizing differential expression analysis, we identified 634 dysregulated genes, using an unadjusted *P* < 0.05 cutoff. Notably, the number of differentially expressed genes (DEGs) was substantially lower than that seen in global *Bmal1-*KO mice, and astrocyte-specific BMAL1 target transcripts such as *Chi3l1* — which is differentially expressed in global KOs (23) — were unchanged in the *Camk2a*-*Bmal1*–KO mice (Figure 4, A and B). Thus, the transcriptome appears quite specific for neuronal *Bmal1* deletion. KEGG pathway gene set enrichment analysis (GSEA) was then performed on the DEGs in this data set, and it identified PD and oxidative phosphorylation as 2 of the top 5 pathways (Figure 4C). Thus, *Bmal1* deletion in neurons causes dysregulation of transcriptional pathways involved in oxidative phosphorylation and PD in cortex.

In order to determine if this gene expression profile in *Bmal1*-KO tissue is relevant to the midbrain, where TH^+^ neuronal loss is observed, we dissected midbrain samples from *CAG*-Cre^ERT2^;*Bmal1*^fl/fl^ mice and Cre^–^ littermate controls 2 months after tamoxifen treatment (5 months old) and performed transcriptomics analysis via bulk RNA-Seq. Unfortunately, the sequencing from 2 samples did not meet quality-control standards, so we ultimately examined 3 Cre^–^ and 2 Cre^+^ samples. However, we performed exploratory analyses on these data to determine if they showed similar trends as the data from *Bmal1*-KO cortex described above. We examined several known DEGs from previous studies of *Bmal1*-KO mouse brain and observed the expected trends in expression of *Nr1d1*, *Dbp*, and *Chi3l1* as would be seen with *Bmal1* deletion (7, 23) (Supplemental Figure 3A). Exploratory differential gene expression analysis identified 2,444 DEGs that met an unadjusted *P* < 0.05 and 371 that reached a multiple-comparisons adjusted *P* < 0.05 (Figure 4D). We first performed KEGG pathway analysis on this preliminary list of 371 DEGs and identified several specific pathways including oxidative phosphorylation, PD, Huntington’s disease, Prion disease, neurodegeneration, ROS, and Alzheimer’s disease (Figure 4E). We also examined the 2,444 gene group with unadjusted *P* < 0.05 with KEGG pathway analysis. Genes that were upregulated in Cre^+^ mice again showed enrichment for transcripts involved in ribosome function, PD, oxidative phosphorylation, and Huntington’s disease (Supplemental Figure 3B). Pathways that were downregulated genes in Cre^+^ were enriched for transcripts related to cGMP/PKG signaling, glutamatergic synapses, and circadian function (Supplemental Figure 3C). Expression of specific transcripts in the PD KEGG pathway are shown in Supplemental Figure 4D. Thus, while not statistically rigorous, our exploratory transcriptomics analysis of global *Bmal1*-KO midbrain suggests that similar pathways are altered as in our analysis of neuron-specific *Bmal1*-KO cortex and that oxidative phosphorylation and PD are prominent.

**Figure 5:**
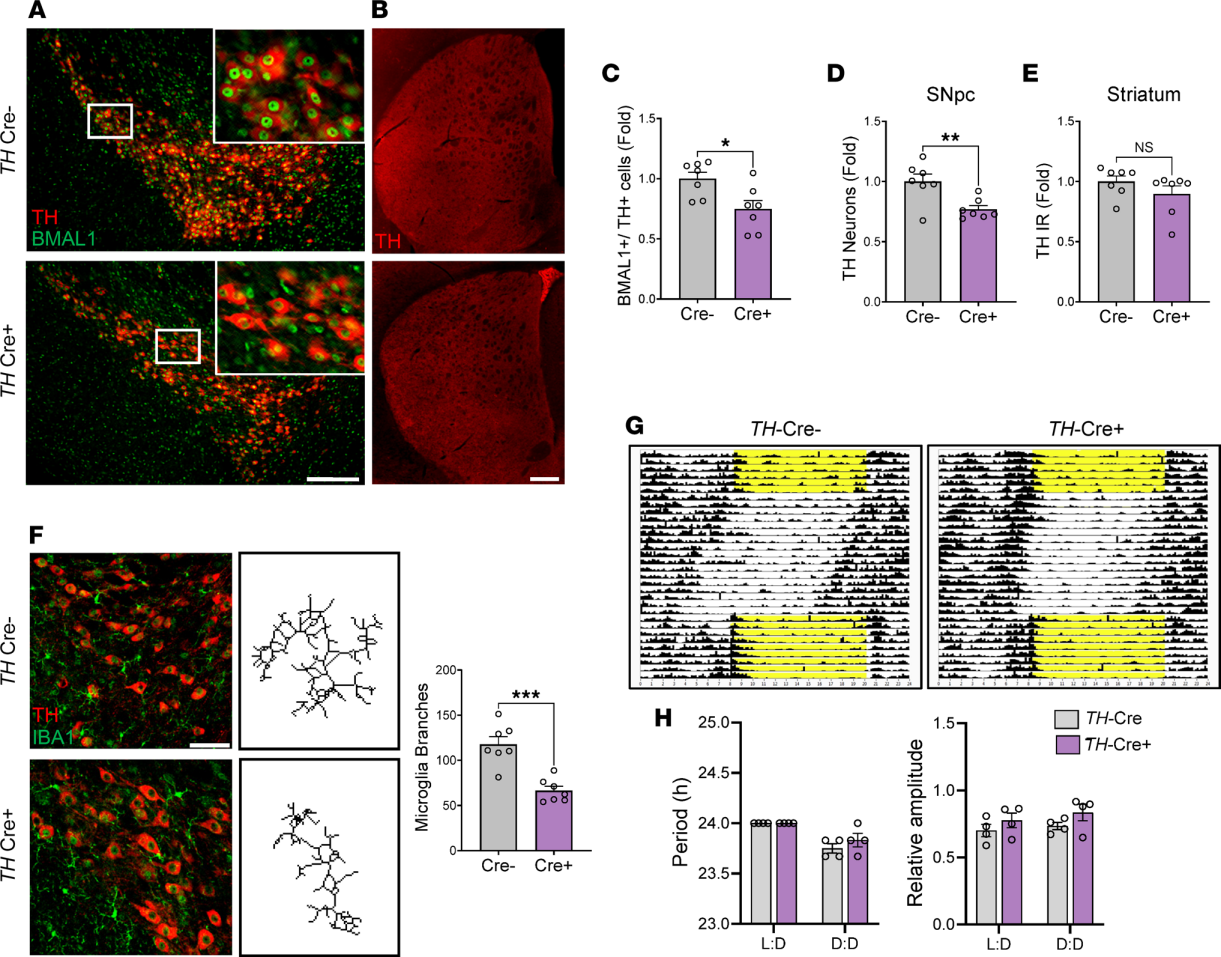
Tyrosine hydroxylase–specific *Bmal1* deletion induces dopaminergic neuron degeneration and increased microgliosis. (**A**) TH (red) and BMAL1 (green) staining in the SNpc of Cre^–^;B*mal*^fl/fl^ control and *TH*-Cre;*Bmal*^fl/fl^ TH-specific *Bmal1*-KO mice. Insets show loss of nuclear BMAL1 staining in a subset of TH^+^ neurons from Cre^+^ mice. Scale bar: 150 μm. (**B**) TH staining in striatum of *TH*-Cre^–^ and Cre^+^ mice. Scale bar: 200 μm. (**C**) Quantification of BMAL1^+^/TH^+^ neurons within the SNpc of Cre^–^ control and Cre^+^ TH-specific *Bmal1*-KO mice. *n* = 6 mice per genotype. (**D**) Stereological counts of TH^+^ cells within the SNpc of Cre^–^ control and Cre^+^ TH-specific *Bmal1*-KO mice. *n* = 6 mice per genotype. (**E**) Quantification of TH immunoreactivity (IR) in striatum, as shown in **B**. *n* = 6 mice per genotype. (**F**) Representative images of TH^+^ neurons (red) and microglia (Iba1, green) within the SNpc of Cre^–^ control and Cre^+^ TH-specific *Bmal1*-KO mice. Representative skeletonized microglia reconstructions are shown in the right panels. Scale bar: 60 μm. Graph indicates average branches per microglial cell within the SNpc of Cre^–^ control and Cre^+^ TH-specific *Bmal1*-KO mice. *n* = 6 mice per genotype, *n* = 9–10 microglia per mouse. (**G**) Representative actrograms showing activity rhythms of *TH*-Cre^–^ and Cre^+^ mice over 32 days. Yellow indicates lights on. (**H**) Quantification of period (left) and relative amplitude (right) from actigraphy in **G**, calculated using data from the 12:12 lights on (L:D) or constant darkness (D:D) periods. For Period and Relative amplitude, no significant main effect of Cre genotype or interaction were observed by 2-way ANOVA. *n* = 5 mice/genotype. For all panels, each circle represents average data from a single mouse. For **C**–**F**, *P < 0.05, **P < 0.01, ***P < 0.001 by 2-tailed *t* test. In all panels, data represent mean ± SEM. All fold changes normalized to average of Cre^–^ condition.

Finally, we sought to determine if deletion of *Bmal1* specifically in TH^+^ neurons was sufficient to induce cell-autonomous degeneration. Accordingly, we generated *TH*-Cre;*Bmal1*^fl/fl^ mice to drive *Bmal1* deletion exclusively in TH^+^ cells. We confirmed by confocal microscopy that BMAL1 protein expression was absent in some but not all TH^+^ cell nuclei (Figure 5, A–C), indicating deletion within a subset of TH^+^ neurons of the SNpc (approximately 25% of cells, on average). The actual deletion of BMAL1 may occur in a higher percentage of cells, since the best antibody we could find gives a slight residual nuclear signal even in germline *Bmal1*-KO mice. However, because of this incomplete deletion, we referred to this as *Bmal1* knockdown (KD), rather than KO. Unbiased stereology revealed that *TH-Bmal1* KD mice showed a 23% reduction of TH^+^ neurons in the SNpc, as compared with Cre^–^ controls, demonstrating that *Bmal1* exerts cell-autonomous effects on dopaminergic neuron survival in vivo (Figure 5D). However, TH immunoreactivity in the striatum was not changed in Cre^+^ mice (Figure 5E). Upon further analysis, we also found a decrease in Iba1^+^ microglia branching within the SNpc in Cre^+^ mice (Figure 5F), indicating a transition to a more amoeboid morphology associated with microglial activation, around the site of TH^+^ neurodegeneration. Microgliosis was not observed in the hippocampus of Cre^+^ mice (Supplemental Figure 4), suggesting a region-specific effect. Notably, we observed no changes in circadian activity rhythms in *TH*-Cre^+^;*Bmal1*^fl/fl^ mice under L:D or constant dark (D:D) conditions (Figure 5, G and H), further demonstrating that the effects are not due to changes in circadian behavioral rhythms.

**Figure 6:**
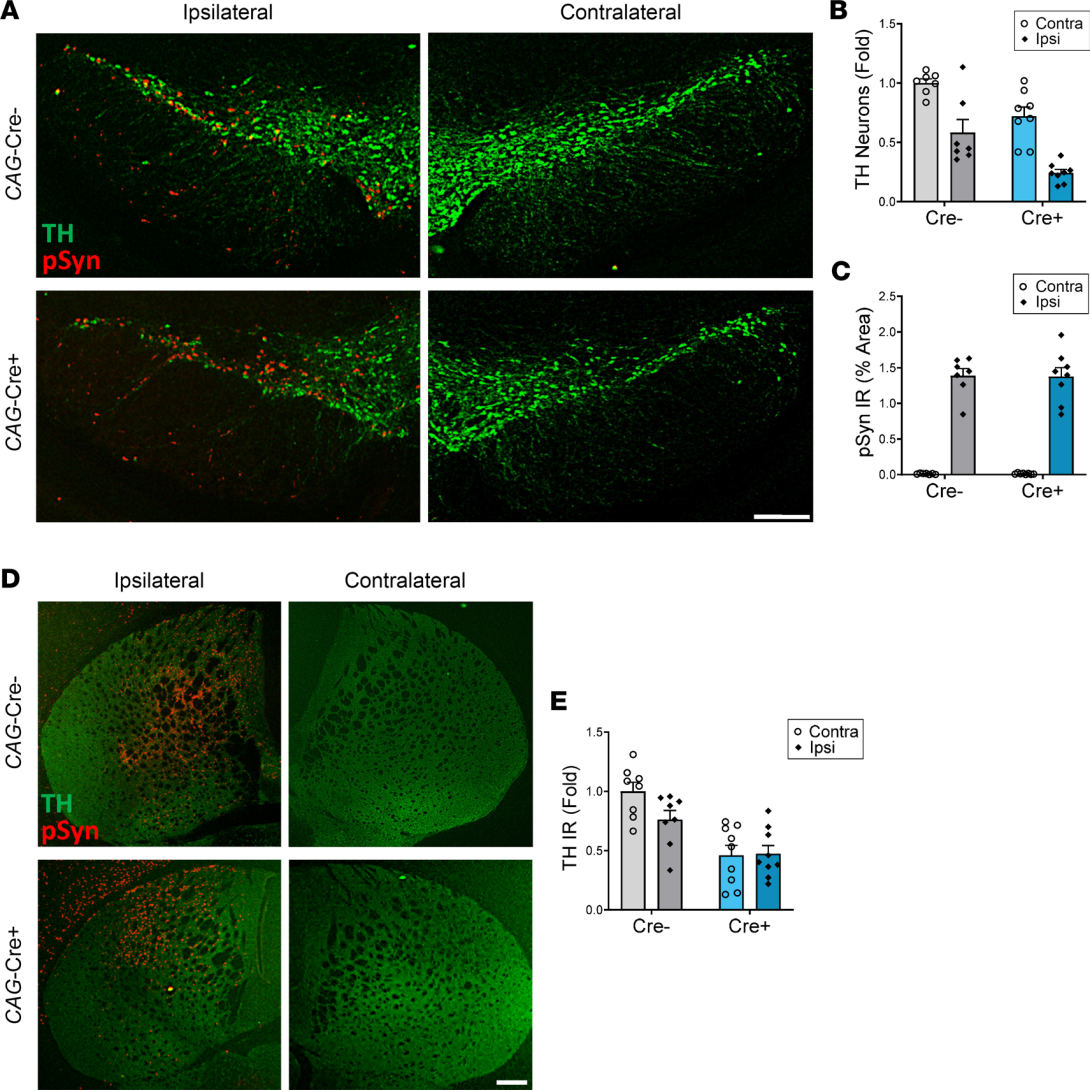
Global *Bmal1*-KO mice does not enhance αSyn-induced dopaminergic neurodegeneration. (**A**) Representative images of TH and phospho-αSyn (pSyn) staining in SNpc of Cre^–^;B*mal1*^fl/fl^ control (*CAG*-Cre^–^) and *CAG*-Cre^+^;*Bmal1*^fl/fl^ global *Bmal1*-KO (*CAG*-Cre^+^) 3-month-old mice. after unilateral striatal injection of αSyn PFFs. Scale bar: 150 μm. (**B**) Quantification of TH^+^ neurons within the ipsilateral and contralateral SNpc of the mice in **A**. *n* = 7–8 mice per genotype, 4 images per mouse. Black diamonds indicate side ipsilateral to PFF injection. Fold change normalized to average of Cre^–^, contralateral condition. Significant main effect of injection side — ipsilateral versus contralateral (F[1,26] = 41.59, *P* < 0.0001) and Cre genotype (F[1,26] = 19.83, *P* = 0.0001) were detected by 2-way ANOVA, but interaction was not significant (F[1,26] = 0.1887, *P* = 0.667). *n* = 7–8 mice/genotype. (**C**) Quantification of pSyn staining (% area) in the SNpc of the mice in **A**. *n* = 7–8 mice per genotype, with 2 images averaged per mouse. Black diamonds indicate side ipsilateral to PFF injection. Main effect of injection side (ipsilateral versus contralateral) was significant (F[1,26] = 271.0, *P* < 0.0001) by 2-way ANOVA, but genotype (F[1,26] = 0.005, *P* = 0.9447) and interaction (F[1,26] = 0.005, *P* = 0.0940) were not significant. (**D**) Representative images of TH (green) and pSyn (red) immunoreactivity (IR) in the striatum of Cre^–^;*Bmal1*^fl/fl^ control (*CAG*-Cre^–^) and *CAG*-Cre^+^;*Bmal1*^fl/fl^ global *Bmal1*-KO (*CAG*-Cre^+^) 3-month-old mice after unilateral striatal injection of α-synuclein PFFs. Scale bar: 200 μm. (**E**) Quantification of striatal TH IR intensity from images in **D**. *n* = 7–8 mice/genotype. Fold change normalized to average of Cre^–^ condition. Main effect of genotype was significant (F[1,30] = 29.13, *P* < 0.0001) by 2-way ANOVA, but main effect of side of injection (F[1,30] = 2.171, *P* = 0.1510) and interaction (F[1,30] = 2.712, *P* < 0.1100) were not significant. In all panels, data represent mean ± SEM, and each circle indicates 1 animal.

A previous study showed that germline *Bmal1* deletion can exacerbate dopaminergic neuron loss caused by the mitochondrial complex I inhibitor MPTP (17). To test this relationship in another model system relevant to PD, we examined TH^+^ neuron survival in *CAG-Bmal1*–KO mice following intrastriatal injection of αSyn preformed fibrils (PFFs). In this model, exogenous αSyn PFFs are injected into the striatum and are likely transported in a retrograde manner to the SNpc, inducing misfolding of endogenous αSyn and causing dopaminergic neurodegeneration (24, 25). We treated mice with tamoxifen at 2 months old, injected αSyn PFFs into the right striatum at 3 months old, and euthanized the mice at 6 months old. We again observed a loss of TH^+^ neurons in the Cre^+^ mice on the contralateral side, demonstrating basal TH neuron loss as a result of *Bmal1* deletion, as seen in Figure 1. αSyn PFF injection caused a similar proportional loss of TH^+^ neurons in the ipsilateral SNpc of Cre^–^ control mice compared with Cre^+^ mice (Figure 6, A and B). Thus, *Bmal1* KO does not seem to exacerbate αSyn PFF–induced TH neuron loss, though Cre^+^ mice ended up with fewer TH^+^ neurons because they had fewer neurons at baseline. We have previously shown that *Bmal1* deletion can prevent αSyn seeding and spreading (8) but observed no difference in pSer129- αSyn IHC staining in the SNpc between genotypes (Figure 6C). This is likely because PFFs enter TH^+^ nerve terminals in the striatum directly after injection and cause SNpc injury, without the need for seeding or spreading. Striatal TH immunoreactivity was decreased in Cre^+^ mice but was not afftected by PFF injection (Figure 6, D and E). We also tested locomotor activity in tamoxifen-treated *CAG*-Cre^ERT2+^;*Bmal1*^fl/fl^ and Cre^–^ controls 80 days after PBS or PFF injection using the pole-descending test, which is sensitive to dopaminergic cell loss (Supplemental Figure 5) (26). While we observed an effect of *Bmal1* genotype on this behavior, there was no effect of PFF injection and no interaction between genotype and PFF treatment. *CAG-Bmal1*–KO mice surprisingly performed better on this motor task compared with Cre^–^ controls, suggesting that their behavioral changes were not driven by loss of dopaminergic neurons. The lack of behavioral effect after PFF injection is presumably because the degree of TH^+^ neuron loss in this particular cohort was not large enough to cause major motor symptoms, as ~80% neuron loss has been suggested as necessary to cause motor deficits (26). It is also notable that unilateral injections, as done here, are less effective than bilateral injections at producing behavioral deficits (27).

A previous study suggests that loss of TH^+^ neurons in global *Bmal1*-KO mice in response to MPTP may be mediated by microglia (17). To determine if any pathological phenotype is mediated by loss of microglial *Bmal1* expression, we employed the same α-syn PFF model using microglia-specific *Bmal1-*KO mice (*Cx3cr1-*Cre^ERT2^*;Bmal1*^fl/fl^). First, no loss of neurons was observed on the contralateral side when comparing Cre^–^ control and Cre^+^ microglial *Bmal1*-KO mice, suggesting that spontaneous TH^+^ neuronal loss is not mediated by *Bmal1*-deficient microglia (Supplemental Figure 6). Second, microglia-specific KO mice showed no difference in TH^+^ cell number or phosphosynuclein staining between genotypes on the ipsilateral side of the injection (Supplemental Figure 6), further supporting a cell-autonomous effect of neuronal *Bmal1* on dopaminergic neurodegeneration.

## Discussion

Our data suggest that the circadian clock transcription factor BMAL1 plays a cell-autonomous role in regulating dopaminergic neuron viability in the SNpc. This effect is not replicated by light-mediated disruption of behavioral circadian rhythms or by glia-specific *Bmal1* deletion. Neuronal BMAL1 regulates transcriptional pathways related to PD and oxidative phosphorylation, and neuronal *Bmal1* deletion leads to spontaneous death of a portion of TH^+^ neurons. Our findings reveal BMAL1 as a contributor to dopaminergic neuron survival and suggest that environmental or disease states that reduce BMAL1 expression might trigger or exacerbate neurodegeneration and parkinsonian phenotypes.

Previous reports have demonstrated that both altered 24-hour light cycle (16) and global germline *Bmal1* deletion (17) can exacerbate TH^+^ neuronal loss in the SNpc in response to the mitochondrial toxin MPTP. Our findings expand upon these observations by using multiple cell- and tissue-specific *Bmal1*–KO mice to show spontaneous TH neuron loss following neuronal *Bmal1* deletion. Loss of microglial *Bmal1* was implicated as the mechanism of degeneration in one of these previous studies, based on cell culture experiments (17). However, our data showing TH^+^ neuron loss in *Nestin-Bmal1*–KO mice (which spares microglial *Bmal1* expression), as well as our observation that microglia-specific *Bmal1* deletion did not alter either basal or αSyn PFF–induced TH^+^ neuronal loss, indicates that microglial *Bmal1* does not drive the neurodegeneration phenotype, at least in our models. Astrocyte-specific *Bmal1* deletion can cause astrogliosis (6), but deletion of *Bmal1* in astrocytes also had no effect on dopaminergic neuronal survival in our experiments. Moreover, we did not observe loss of TH^+^ neurons after 3 months of light-induced circadian desynchrony (T20 cycle). Because numerous light-based circadian desynchrony models exist, each with potentially unique effects, further study is needed to understand the effect of altered light cycles on dopaminergic neurodegeneration. However, our data point strongly to neuronal loss of *Bmal1* as being fundamental to this TH neuronal death phenotype, independent of changes in behavioral circadian rhythms. While this effect of *Bmal1* deletion may be independent of its role in circadian rhythms, we cannot exclude the possibility that disruption of cell-intrinsic circadian rhythms in TH neurons via *Bmal1* deletion contributes to cell death.

Our findings build on previous studies from our laboratory, suggesting that BMAL1 plays a role in neuronal redox homeostasis. Global *Bmal1*-KO mouse cortex shows increased lipid peroxidation and impaired circadian expression of redox defense transcripts such as *Nqo1* and *Aldh2* (7) as well as diminished expression of several glutathione S-transferase transcripts (6). Our RNA-Seq analysis shows that BMAL1 regulates transcripts related to PD pathogenesis and oxidative phosphorylation in midbrain tissue from global *Bmal1*-KO mice and in cortical tissue from neuron-specific *Bmal1*-KO mice. Dopaminergic neurons of the SNpc are uniquely sensitive to oxidative stress and mitochondrial dysfunction, and this may explain why neuronal loss was noted in the SNpc but not in the hippocampus or other regions in *Bmal1*-KO mice. Accordingly, we previously demonstrated that, while *Bmal1*-deficient cortical neuron cultures were sensitized to hydrogen peroxide–induced cell death, global *Bmal1* deletion did not affect basal neuronal survival in the cortex and striatum in vivo. However, hemizygous deletion of *Bmal1* sensitized striatal neurons to death induced by the mitochondrial toxin 3-nitropropionic acid (7). Thus, *Bmal1* deletion may render all neurons more vulnerable to oxidative stress, which could specifically manifest as TH neuron loss.

Our findings emphasize that the effects of BMAL1 in neurological disease models are highly cell type and context dependent. Global *Bmal1* deletion has previously been shown to cause brain oxidative stress and synapse loss (7), exacerbate MPTP-induced dopaminergic neuron loss(17), accelerate amyloid plaque formation (18), and lower seizure threshold (28, 29) — all effects that should promote neurodegeneration. Conversely, previous studies show that global *Bmal1* deletion can mitigate stroke severity (9) and spinal cord injury(10). We have previously shown that *Bmal1* deletion specifically in astrocytes induces astrogliosis (6) and can prevent both tau and αSyn aggregation in vivo, in part through regulation of the chaperone protein BAG3 or through changes in lysosomal function (8, 30). Similarly, other groups have demonstrated that deletion of *Bmal1* in microglia appears to reduce inflammatory activation and reduce damage caused by ischemic stroke (31). Deletion of *Bmal1* in either lymphocytes or myeloid cells has also been shown to also reduce disease severity in the experimental autoimmune encephalomyelitis (EAE) model of multiple sclerosis (32, 33). Our data in this manuscript showing loss of TH neurons following global or neuronal *Bmal1* deletion (Figure 1, Figure 2, and Figure 4) are consistent with our previous study showing that neuronal *Bmal1* deletion sensitizes neurons to oxidative stress and death (7), while *Bmal1* deletion in glia may have more variable (and often protective) effects (8). Thus, BMAL1 appears to be important for neuronal survival in general. Further study into the cell- and model-dependent effects of *Bmal1* and other clock genes is needed to fully understand how core clock disruption may influence neurodegenerative diseases.

Sleep and circadian disturbances are common in synucleinopathies including PD. Several human studies show alterations in the expression of clock gene transcripts in PBMCs from patients with PD. *Bmal1* transcript levels in PBMCs were suppressed in patients with PD in 2 studies (14, 15), while the rhythmic *Bmal1* expression was blunted in patients with PD in another (13). While it is unknown how peripheral blood and SNpc *Bmal1* expression are correlated in patients with PD, our studies suggest that environmental factors and disease processes that affect circadian gene expression in the brain, in particular those that lead to suppressed *Bmal1* expression, could put dopaminergic neurons at risk of death. Conversely, therapies that augment *Bmal1* expression might be expected to protect TH^+^ neurons, and this possibility warrants future investigation.

In summary, the circadian clock gene *Bmal1* exerts cell-autonomous effects on dopaminergic neuronal survival. Neuronal *Bmal1* regulates transcripts related to oxidative phosphorylation and PD, and loss of neuronal *Bmal1* leads to death of TH^+^ neurons in the SNpc. These effects are not phenocopied by light-induced circadian desynchrony or by glial *Bmal1* deletion. Our results provide insights into the relationship between the neuronal circadian clock and PD pathogenesis.

## Methods

### Mice.

*Bmal1*^fl/fl^, *CAG*-Cre^ERT2^, *TH*-Cre, *Aldh1l1*-Cre^ERT2^, *Cx3cr1*-Cre^ERT2^, and *Nestin*-Cre mice were all originally obtained from The Jackson Laboratory and were crossed in our colony. *CaMK2a*-iCre;*Bmal1*^fl/fl^ were previously described (22, 34), and fixed/frozen tissue were prepared in house. All mice were maintained on a C57BL/6J background. All Cre^ERT2^ lines were treated with tamoxifen (MilliporeSigma) at 2 months of age. Mice were given daily i.p. injections of tamoxifen in corn oil at a dose of 2 mg/day for 5 consecutive days. Both Cre^+^ and Cre^–^ mice were treated with tamoxifen. In all cases, Cre^+^ mice were hemizygous for Cre. Male and female mice were used in all experiments, as we have not observed sex differences in any of the measured endpoints.

### IHC.

Mice were deeply anesthetized with pentobarbital, followed by transcardiac perfusion with cold PBS + 5% heparin for 3 minutes. Brains were then removed and immersion fixed in 4% paraformaldehyde for 24 hours at 4°C; they were then incubated with 30% sucrose solution for at least 48 hours at 4°C. Fixed, frozen brains were sectioned on a Leica sliding microtome to generate 40 μm coronal sections, which were stored at –20°C in cryoprotectant solution containing 30% ethylene glycol, 15% sucrose, and 15% phosphate buffer. The following antibodies were used: TH (MilliporeSigma, AB152, 1:2000), BMAL1 (Novus, NB100-2288, 1:2000), Iba1 (Abcam, AB5076, 1:500), NeuN (MilliporeSigma, MAB377, 1:2000), phospho–serine 129 αSyn (pSer129- αSyn) (BioLegend, 825704, 1:1000). Sections were washed 3 times for 5 minutes each in TBS before being blocked and permeabilized in TBS containing 0.25% Triton X-100 (TBSX) containing on 3% goat or donkey serum (Abcam) at room temperature with shaking, followed by overnight incubation with primary antibody diluted in TBSX containing 1% goat or donkey serum at 4°C overnight with shaking. The sections were then washed 3 times for 5 minutes each in TBSX and were then incubated with secondary antibodies for 2 hours at room temperature with shaking. For immunofluorescence microscopy, sections were incubated with fluorescently labeled secondary antibodies at a 1:1,000 dilution in TBSX with protection from light; they were then washed in TBSX and mounted. For DAB-stained sections, a biotinylated secondary antibody (Vector, BA-1000) was used at 1:1,000, and sections were then washed 3 times in TBSX. The tissue was then incubated with HRP-streptavidin (Vectashield) for 2 hours in TBS at room temperature with shaking. Sections were then washed again with TBS for 3 times for 5 minutes each with shaking and were then incubated with diaminobenzidine solution with 0.25% of 4% nickel chloride and 0.05% of 30% hydrogen peroxide for 2–5 minutes. The tissue was then mounted and dried for 24 hours, washed in water twice for 2 minutes, and incubated in cresyl violet solution for 2 minutes. Tissue was then serially dehydrated in ethanol and xylene before being coverslipped. Sections were mounted on slides and coverslipped with Prolong Gold mounting reagent.

### IHC quantitation.

Epifluorescence and bright-field images were taken with a Keyence BZ-X810 microscope at 10× magnification. Using ImageJ software (NIH), regions of interest were manually traced, and digital intensity was thresholded to encompass all specific immunoreactivity, with this threshold value maintained for all sections. All regions of interest were traced, thresholded, and analyzed in a blinded manner. Percent area coverage was then calculated for each image. In general, 1 image from each section and 2 sections/mouse were averaged, and the average from each mouse was used as a data point. Four to 5 sections per mouse were counted, with 240 μm between them.

### Morphologic estimation of TH^+^ neuron number.

For some experiments in Figure 5, fluorescence images of TH IHC were captured with a Keyence BZ-X810 microscope at 10× magnification. Using ImageJ, regions of interest were drawn around the SNpc, images were subjected to color correction, and digital intensity was thresholded so that somata of TH^+^ neurons appeared as single objects. The thresholded images were then analyzed using the “analyze particles” command in ImageJ. The size (pixel^2^) function was then corrected to include particles from 10 to 800 pixels^2^. The default circularity was maintained between 0–1 arbitrary units. Images were then converted to outlines, and the output value titled as “counts” were recorded as the number of particles in the image. Four 40 μm SNpc sections from each mouse were included for the analysis, and each section was 240 μm apart from one another. The sections were picked from bregma –2.78 mm to bregma –3.68 mm. The average of the 4 values were used to determine the number of TH^+^ particles for a given mouse.

### Unbiased stereology.

Stereologic estimation of TH^+^ neurons in the SNpc was performed using a Zeiss AxioImager.M2 microscope and StereoInvestigator software (MBF Bioscience). Unbiased optical fractionator estimates of the total number of TH^+^ cells within the SNpc were generated using published methods (35, 36). Briefly, sections were obtained by randomly choosing the first section, followed by every fifth section thereafter. The counting frame size for all experiments was 90 μm by 80 μm, the grid size was 150 × 150, and the optical dissector height used was 18. Gundersen coefficients were less than or equal to 0.1 (m = 0). One hemisphere was analyzed per mouse, and all assessments were performed in a blinded manner.

### Skeletonization.

Images of Iba^+^ cells were acquired from the SNpc using a Zeiss LSM 800 confocal microscope. Morphological analysis of confocal images was acquired using the protein marker Iba1. Images were taken at 20× and were analyzed using ImageJ software (NIH). The images were modified as 8-bit and *Z* stack projection images; then, the resulting images were smooth processed, binarized, and skeletonized, using the Skeletonize Plugin. A mask of the images was then created using the particle analysis function to subtract from skeletonized images. The resulting images were processed using the Analyze Skeleton 2D/3D option in the Skeletonize Plugin, and the number of branches per cell was obtained from the results tables. Three sections from each mouse were used for this experiment, and 1–2 images from each section were taken. Three Iba1^+^ microglia were randomly chosen from each image, resulting in 10 analyzed microglia for each mouse. The average of all 10 microglial branching outputs was used as a data point for each mouse.

### αSyn fibril preparation and stereotactic injection.

Purification of recombinant mouse sequence αSyn monomer and in vitro fibril assembly were performed as described with minor modifications (37, 38). Briefly, αSyn monomer was produced in BL21 *E. coli* and purified by size exclusion and anion exchange chromatography. Endotoxin was removed using Pierce High Capacity endotoxin removal columns. Fibril assembly reactions were carried out by shaking αSyn monomer at 5 mg/μL in an Eppendorf ThermoMixer at 1,000 rpm for 7 days. An aliquot of the resulting suspension was centrifuged at 15,000*g* for 20 minutes, and the PFF concentration was estimated by subtracting the concentration of αSyn monomer in the resulting supernatant from the starting monomer concentration. PFFs were adjusted to 5 mg/μL, aliquoted, and stored at −80°C until use. Before use, aliquots were thawed and sonicated briefly in a Qsonica water bath sonicator. Mice at approximately 3 months old were anesthetized with isoflurane and injected with 1 μL of αSyn PFF suspension (5 μg) into the left dorsal striatum (0.2 mm anterior and 2.0 mm lateral to bregma and 3.2 mm below the surface of the skull) using a Hamilton microsyringe attached to a motorized injector (Stoelting). At 3 months after injection, animals were anesthetized with pentobarbital followed by perfusion for 2 minutes with heparinzed PBS. Whole brains were removed and fixed in 4% paraformaldehyde for 48 hours; they were then transferred to a 30% sucrose solution for another 48 hours before sectioning. Coronal sections (40 μm) were taken from whole brains and stored in cryoprotectant for later use.

### Actigraphy.

For actigraphy studies, mice were single housed in ventilated, temperature-controlled, and light-controlled circadian cabinets with white LED lighting at a 800–1,000 lux max (Actimetrics). Cabinets were housed in a dedicated mouse circadian room kept quiet and in dim red light at all times. Mouse activity was monitored continuously using wireless infrared motion sensors mounted to each cage, with data collected using ClockLab Wireless data collection software and visualized using ClockLab analysis software (Actimetrics). Lighting was controlled by computer. Cage changes were performed every 2 weeks under red light conditions.

### Pole test.

The pole descending test has been used to assess locomotion deficits related to impairment of basal ganglia function in mice (25). Mice were placed with their head facing up at the top of a knurled vertical metal pole 57 cm long and 1.25 cm in diameter. The time required for mice to turn to face downward and the total time to descend to the base of the pole were measured. Mice were acclimated to the testing room and were trained with 3 successive trials over 3 days. On the day of testing, each mouse was evaluated in 3 separate trials, and the fastest time for each mouse was recorded. A cutoff time of 120 seconds was used for mice that did not reach the bottom.

### Transcriptomics analysis.

Total mRNA was isolated from frozen cerebal cortex or dissected midbrain samples as previously described (7). Briefly, tissue was submerged in Trizol reagent (Thermo Fisher Scientific) and mechanically dissociated using a bead homogenizer. Chloroform extraction was performed; the supernatant was collected and mRNA was isolated using Ambion PureLink RNA iolation kit (Thermo Fisher Scientific), following the manufacturers instructions. RNA-Seq and analysis were performed by the Genome Technology Access Center at Washington University using their standard methods, which are summarized here: Sample RNA integrity was determined using a Tapestation, and library preparation was performed with 10 ng of total RNA for samples with a Bioanalyzer RIN score greater than 8.0. Double-stranded cDNA was prepared using the SMARTer Ultra Low RNA kit for Illumina Sequencing (Takara-Clontech) per manufacturer’s protocol. cDNA was then fragmented using a Covaris E220 sonicator (at peak incident power 18, duty factor 20%, cycles per burst 50 for 120 seconds). The cDNA was blunt ended, had an A base added to the 3′ ends, and then had Illumina sequencing adapters ligated to the ends. Ligated fragments were then amplified for 12–15 cycles using primers incorporating unique dual index tags. The fragments for each sample were then pooled in an equimolar ratio and sequenced on an Illumina NovaSeq-6000 using 150 bp paired end reads. Basecalls and demultiplexing was performed with Illumina’s RTA 1.9 software, and the reads were aligned to the Mus musculus Ensembl release 76 GRCm38 primary assembly with STAR version 2.5.1a. Gene counts were quantitated with Subread:featureCount version 1.4.6-p5.

All gene counts were then imported into the R/Bioconductor package EdgeR, and TMM normalization size factors were calculated to adjust the samples for differences in library size. Ribosomal genes were removed, and only genes expressed greater than 1 count per million in at least 4 samples were kept for further analysis. The adjusted TMM size factors and the matrix of counts were then imported into the R/Bioconductor package Limma. Weighted likelihoods were then calculated for all samples, and the count matrix was transformed to moderated log_2_ counts per million with Limma’s voomWithQualityWeights.

Differential gene expression was calculated using R package DESeq2. A volcano plot was constructed using the package EnhancedVolcano, with a fold-change cutoff of 1.5 and a *P* value cutoff of 0.05. KEGG pathway GSEA was performed using R package clusterProfiler and enrichplot.

### Statistics.

Data were analyzed using GraphPad Prizm 9.1.0. For all analyses, *F* test was performed to compare variances between groups. If the *F* test was not significant, a 2-tailed, unpaired Student’s *t* test was performed on data sets with 1 variable, or 2-way ANOVA was used for 2 variable data sets. For 2-way ANOVA, Tukey’s multiple comparisons test was used to examine significant main effects/interactions. If the *F* test showed significant difference in variance between groups, then a nonparametric test was used (Mann-Whitney *U* test for 1 variable, Kruskal-Wallis test for 2 or more variables). In all figures, data are shown as mean ± SEM.

### Study approval.

All mouse studies were approved by the Washington University IACUC, which is accredited by the American Association for Accreditation of Laboratory Animal Care (AAALAC). Studies were conducted under supervision of the Washington University Department of Comparative Medicine in accordance with approved protocols.

### Data availability.

All mouse lines are commercially available through The Jackson Laboratory. RNA-Seq data sets are publicly available on the NIH GEO website. Raw RNA-Seq data from midbrain of global *Bmal1-*KO mice are available under GEO accession no. GSE241200. RNA-Seq data from the cortex of neuron-specific *Bmal1-*KO mice are available under GEO accession no. GSE241218. Raw data from all graphs are available in the Supporting Data Values document.

## Author contributions

MKK and PWS share first-author position. MKK was listed first because he generated a larger number of the figures, and PWS agreed to this arrangement. AAD and ESM share corresponding author position, as AAD was responsible for overseeing TH stereology and synuclein models, while ESM managed all aspects of mouse model generation and RNA-Seq, with both authors sharing conceptual and editorial input. ESM is listed last because both first authors were supported and managed by him in his lab. Experiments were conceived and designed by MKK, PWS, AAD, and ESM. Mouse breeding, tissue preparation, IHC, data acquisition, and analysis were performed by MKK, PWS, JNH, PGG, AD, ZMW, BMF, and CJN. JDC and HRN oversaw unbiased stereological analyses. MI and JST generated pan-neuronal *Bmal1*-KO mice and supplied tissue from them. Intrastriatal injections were performed by AAD. MKK, PWS, AAD, and ESM wrote and edited the manuscript. All authors read and approved the final manuscript.

## Supplementary Material

Supplemental data

Supporting data values

## Version 1 11/30/2023

In-Press Preview

## Version 2 01/23/2024

Electronic publication
