# Neuronal deletion of the circadian clock gene *Bmal1* induces cell-autonomous dopaminergic neurodegeneration

**DOI:** 10.1172/jci.insight.181596

**Published:** 2024-08-08

**Authors:** Michael F. Kanan, Patrick W. Sheehan, Jessica N. Haines, Pedro G. Gomez, Adya Dhuler, Collin J. Nadarajah, Zachary M. Wargel, Brittany M. Freeberg, Hemanth R. Nelvagal, Mariko Izumo, Joseph S. Takahashi, Jonathan D. Cooper, Albert A. Davis, Erik S. Musiek

Original citation *JCI Insight*. 2024;9(2):e162771. https://doi.org/10.1172/jci.insight.162771

Citation for this corrigendum: *JCI Insight*. 2024;9(15):e181596. https://doi.org/10.1172/jci.insight.181596

The authors recently became aware of an inadvertent error in presentation of representative TH- and cresyl violet–stained images in Supplemental Figure 2C. The correct panels are shown below. The authors confirm that the quantification is correct and the conclusions of the study are not affected.

The Supplemental data has been updated online.

The authors regret the error.

## Supplementary Material

Supplemental data

## Version 1 08/29/2024

Electronic Publication

