# Supplementary material for: Neuronal deletion of the circadian clock gene Bmal1 induces cell-autonomous dopaminergic neurodegeneration
Source: JCI Insight. 2024 Aug 8;9(15):e181596. doi: 10.1172/jci.insight.181596 (PMC11383828; doi:10.1172/jci.insight.181596)
Supplement: Supplemental data [file jciinsight-9-181596-s268.pdf]

FIGURE S1

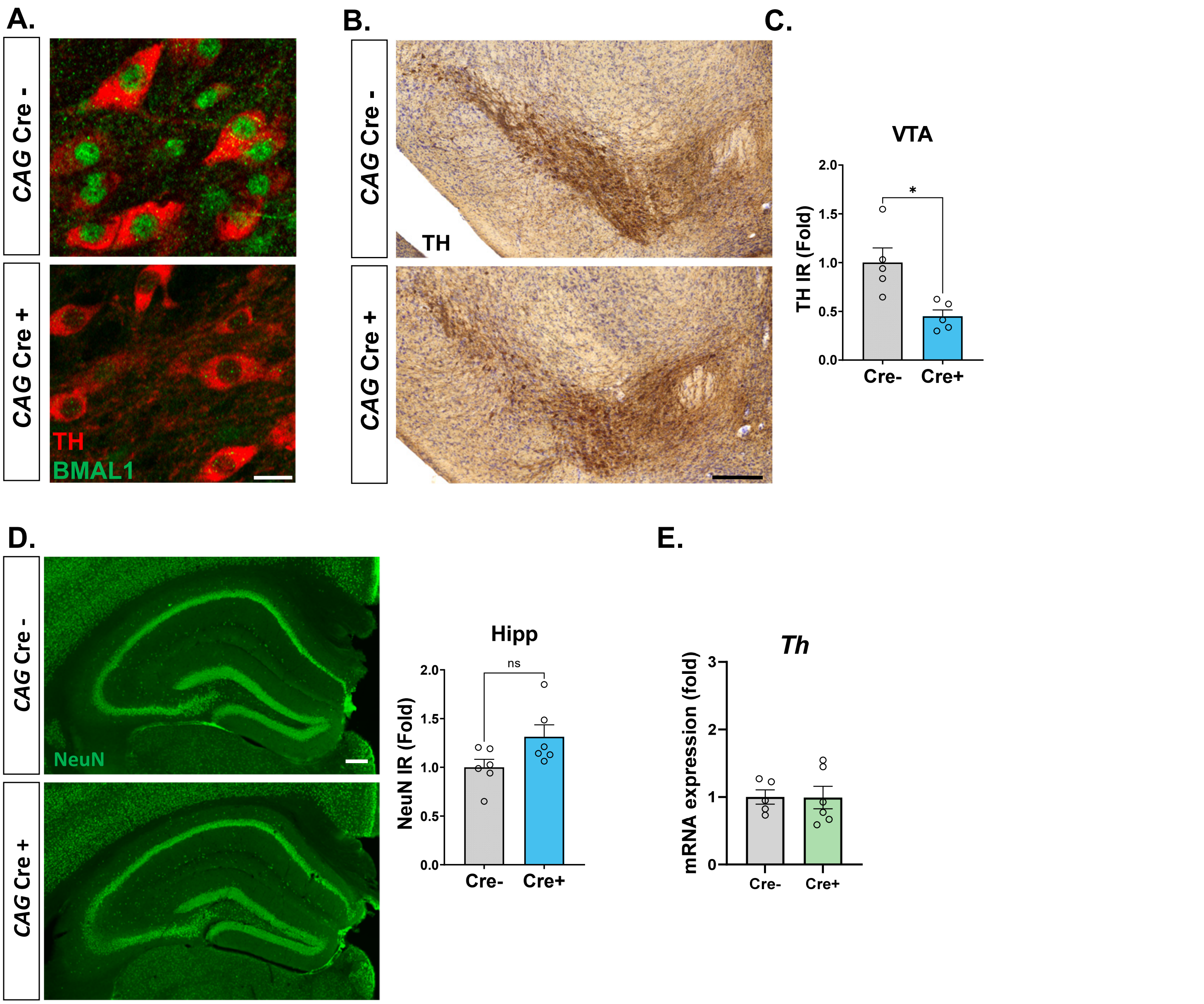

Supplemental Figure 1: Global inducible *Bmal1* KO mice exhibit dopaminergic neuron loss within the Ventral Tegmental Area but does not impact the hippocampus or *Th* gene expression.

A. Representative confocal images of TH (red) and BMAL1 (green) immunoreactivity in the SNpc of global inducible *Bmal1* KO mice (CAG-Cre+) and Cre- control mice, 2 months after tamoxifen. Scale bar = 25µm.

B.Representative images of TH (brown) and cresyl violet (purple) staining of the SNpc and VTA from global inducible *Bmal1* KO mice (CAG-Cre+) and Cre- control mice, 2 months after tamoxifen.

C. Quantification of TH+ neurons in the VTA of the mice from Fig. 1A (shown in B). n=5 mice per genotype. Fold change is normalized to Cre- condition. *$P < 0.05$ by two-tailed t test.

D. Representative images of NeuN staining in the hippocampus of global inducible *Bmal1* KO mice (CAG Cre+) and Cre- control mice. Percent area was used to determine neuronal density and normalized to Cre- condition. Quantification is shown in the graph on the right. n=6 mice per genotype. ns, not significant by two-tailed t test.

E. Quantification of *Th* mRNA in cortex samples from 9mo *Nestin*-Cre;*Bmal1*$^{f/f}$ mice and Cre- controls. No significant difference by 2-tailed t-test ($P>0.1$). Graphs depict mean$\pm$SEM.

FIGURE S2

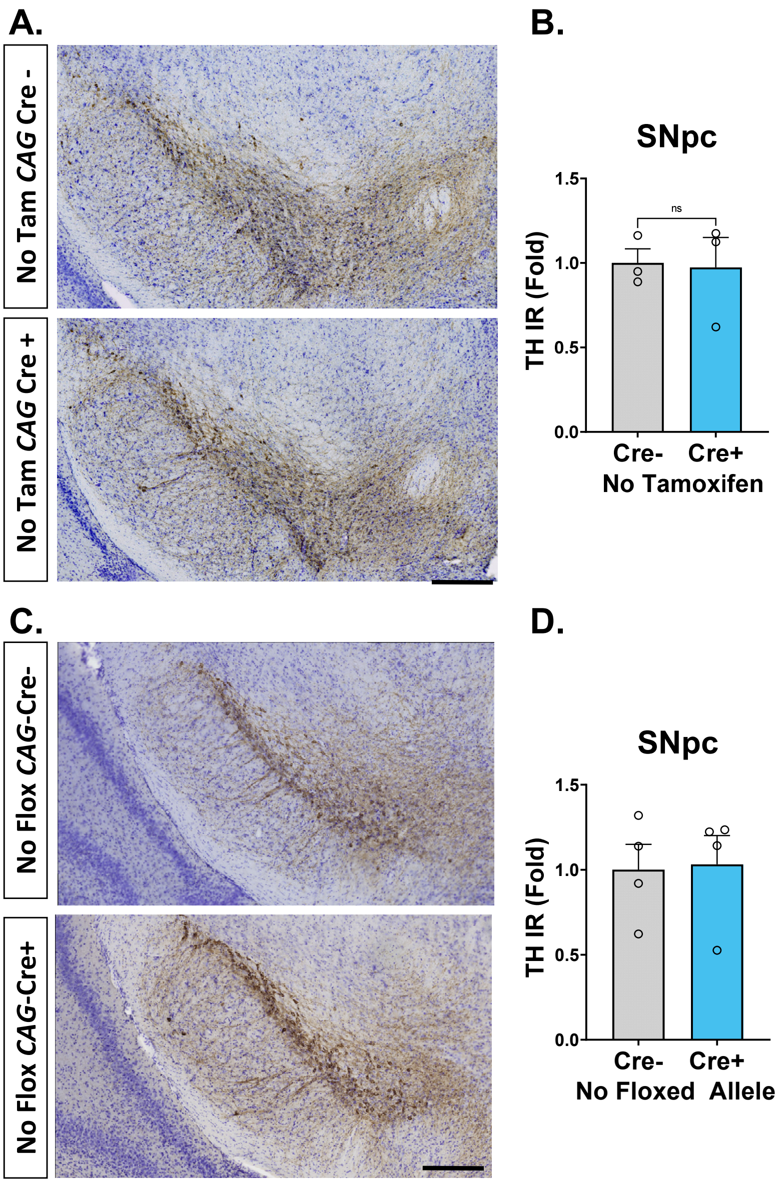

Supplemental Figure 2: Cre expression does not cause dopaminergic neuron loss.

A.Representative images of TH (brown) and cresyl violet (purple) staining the SNpc of *CAG*-Cre^{ERT2+};*Bmal1*$^{f/f}$ and Cre- control mice which were not treated with tamoxifen. Scale bar = 150μm.

B.Quantification of TH+ neurons in the SNpc in mice from A. n=3 mice per genotype. ns, not significant by two-tailed t test (P>0.1).

C.Representative images of TH (brown) and cresyl violet (purple) staining the SNpc of *CAG*-Cre^{ERT2+} and Cre- control mice which were treated with tamoxifen, but did not have floxed alleles. Scale bar = 150μm.

D.Quantification of TH+ neurons in the SNpc in mice from C. n=4 mice per genotype. ns, not significant by two-tailed t test (P>0.1).

Each circle represents data from a single mouse. Fold change was normalized to average of Cre- condition. Graphs depict mean$\pm$SEM.

FIGURE S3

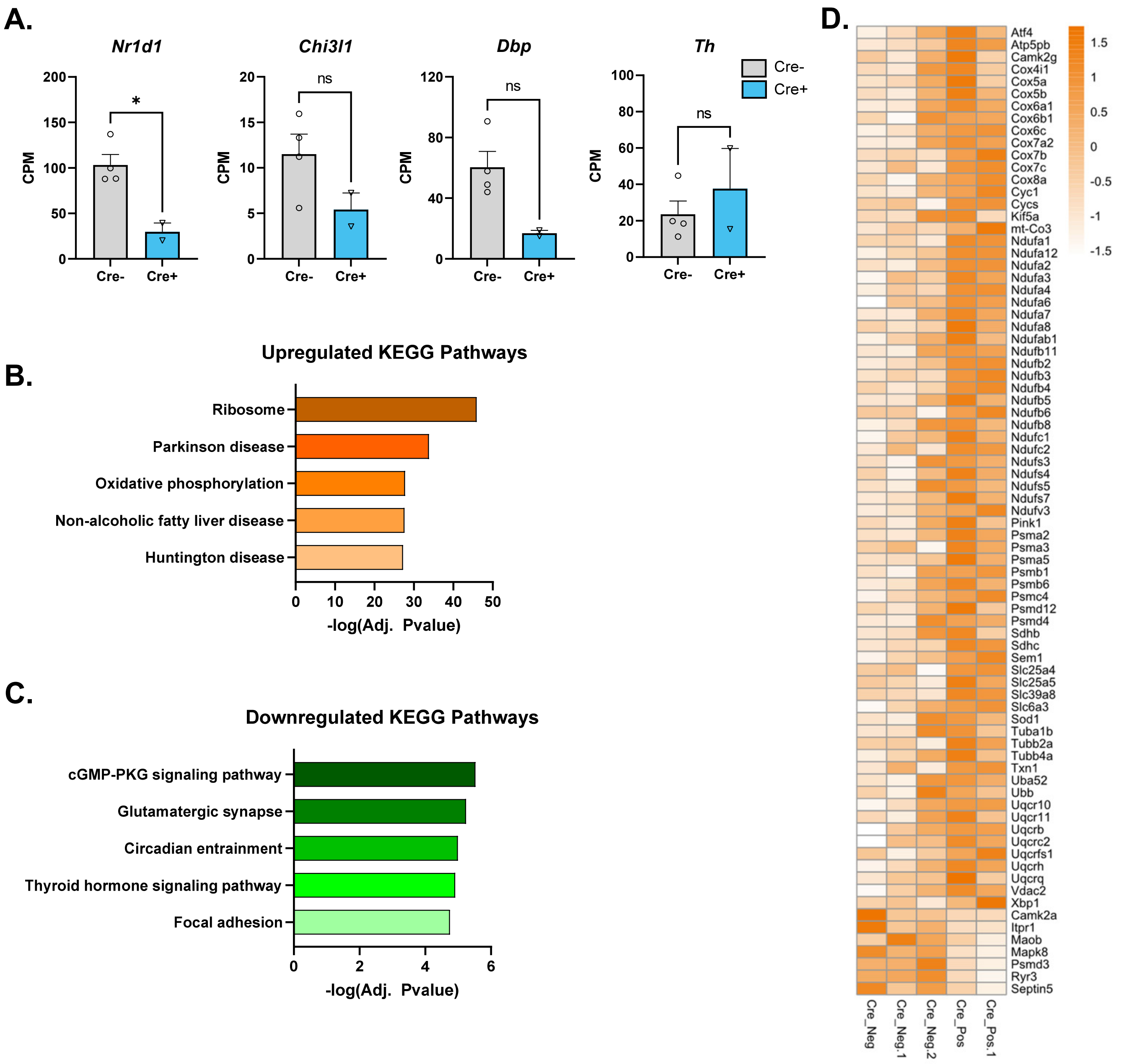

Supplemental Figure 3: Gene expression in midbrain of global *Bmal1* KO mice.

A. Quantification of circadian gene expression (CPM: counts per million) in midbrain tissue from global inducible *Bmal1* KO (*CAG*-Cre+;*Bmal1*$^{f/f}$) and Cre- control mice from RNAseq data.

B. Exploratory KEGG Pathway analysis showing pathways associated with upregulated genes in the *CAG*-Cre+;*Bmal1*$^{f/f}$ midbrain tissue. DEGs with unadjusted p value<0.05 were used.

C. Exploratory KEGG Pathway analysis showing pathways associated with downregulated genes in the *CAG*-Cre+;*Bmal1*$^{f/f}$ midbrain tissue. DEGs with unadjusted p value<0.05 were used.

D. Heatmap showing expression of KEGG Parkinson Disease pathway genes in midbrain tissue from global inducible *Bmal1* KO (*CAG*-Cre+;*Bmal1*$^{f/f}$) and Cre- control mice from RNAseq data.

Graphs depict mean±SEM

FIGURE S4

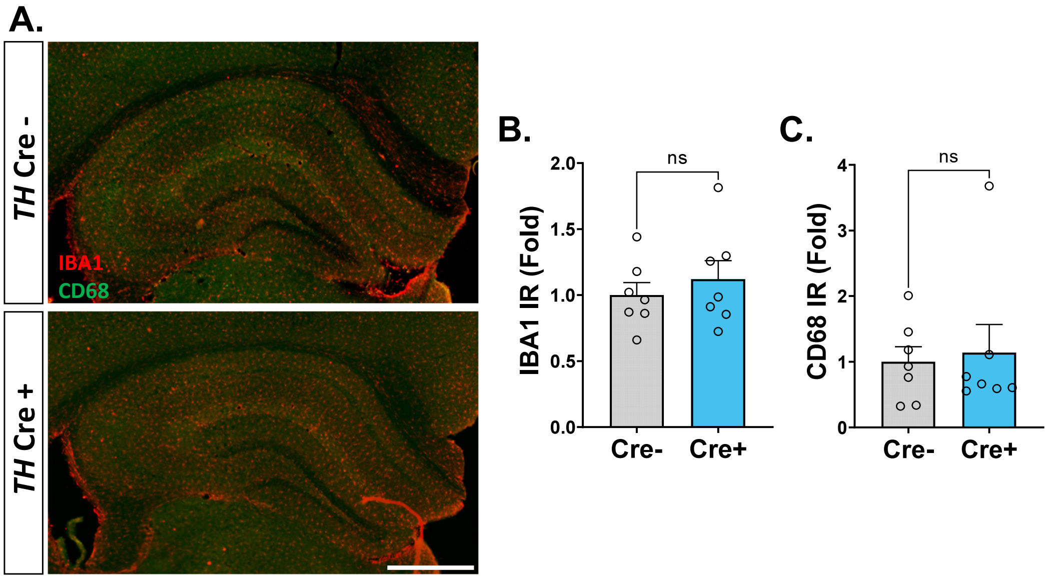

Supplemental Figure 4: Tyrosine Hydroxylase-specific *Bmal1* KO mice exhibit no changes in hippocampal microgliosis.

A. Representative images of IBA1 and CD68 staining in 2-3 month old Cre- control and *TH*-Cre+; *Bmal1*$^{f/f}$ mouse hippocampus.

B. Quantification of IBA1 immunoreactivity (IR) in hippocampus. n=6 mice per genotype, 2-3 sections averaged per mouse.

C. Quantification of CD68 staining. n=6 mice per genotype. ns, not significant by two-tailed t test. 2-3 sections averaged per mouse.

In B and C, fold change was normalized to the average of the Cre- condition. NS = not significant, p>0.1 by 2-tailed T-test. Scale bar = 500µm. Graphs depict mean±SEM.

FIGURE S5

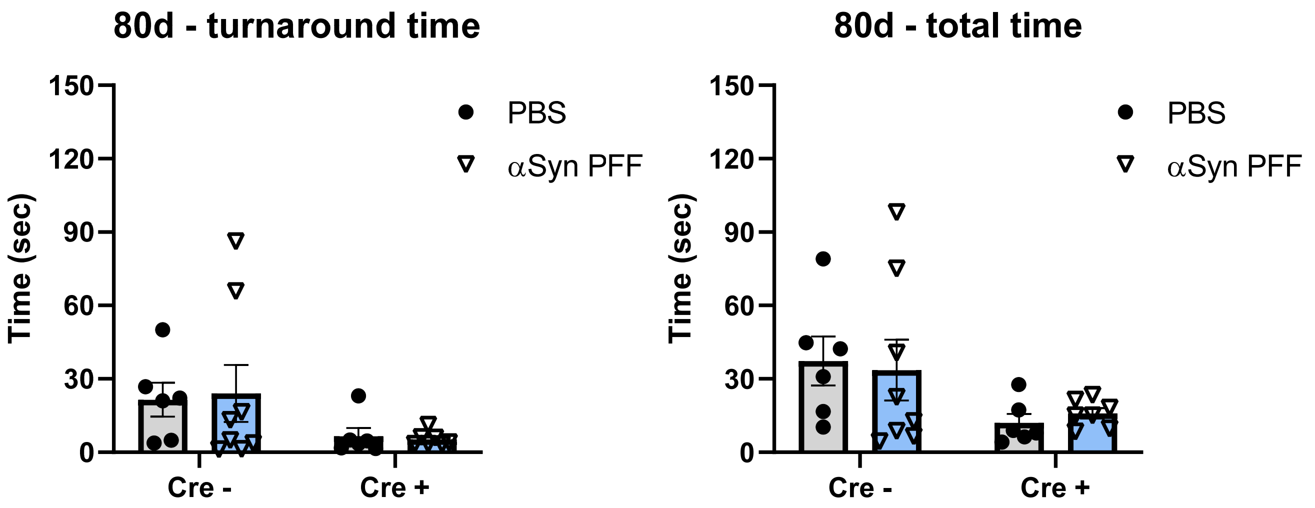

Supplemental Figure 5: Effect of global *Bmal1* deletion and α-syn PFF injection on pole climbing motor behavior. *CAG-Bmal1* KO and Cre- control mice were treated as in Fig. 4 with tamoxifen at 2mo, then with unilateral striatal injection of PBS or α-syn PFFs at 3 mos. 80 days later, mice were tested in pole climbing, with both turnaround time (left panel) and total climb time (right panel) recorded. 2-way ANOVA showed a significant main effect of genotype, but no effect of α-syn PFF injection, and no interaction between genotype and α-syn PFF injection.

For turnaround time, 2-way ANOVA showed a significant main effect of Cre genotype ($F(1,23)=4.593$, $P=0.0429$), but main effect of PFF injection ($F(1,23)=0.007644$, $P=0.9311$) and interaction $F(1,23)=0.06145$, $P=0.8064$) were not significant.

For total time time, 2-way ANOVA showed a significant main effect of Cre genotype ($F(1,23)=5.745$, $P=0.0$), but main effect of PFF injection ($F(1,23)=0.0001$, $P=0.9968$) and interaction $F(1,23)=0.1787$, $P=0.6764$) were not significant.

FIGURE S6

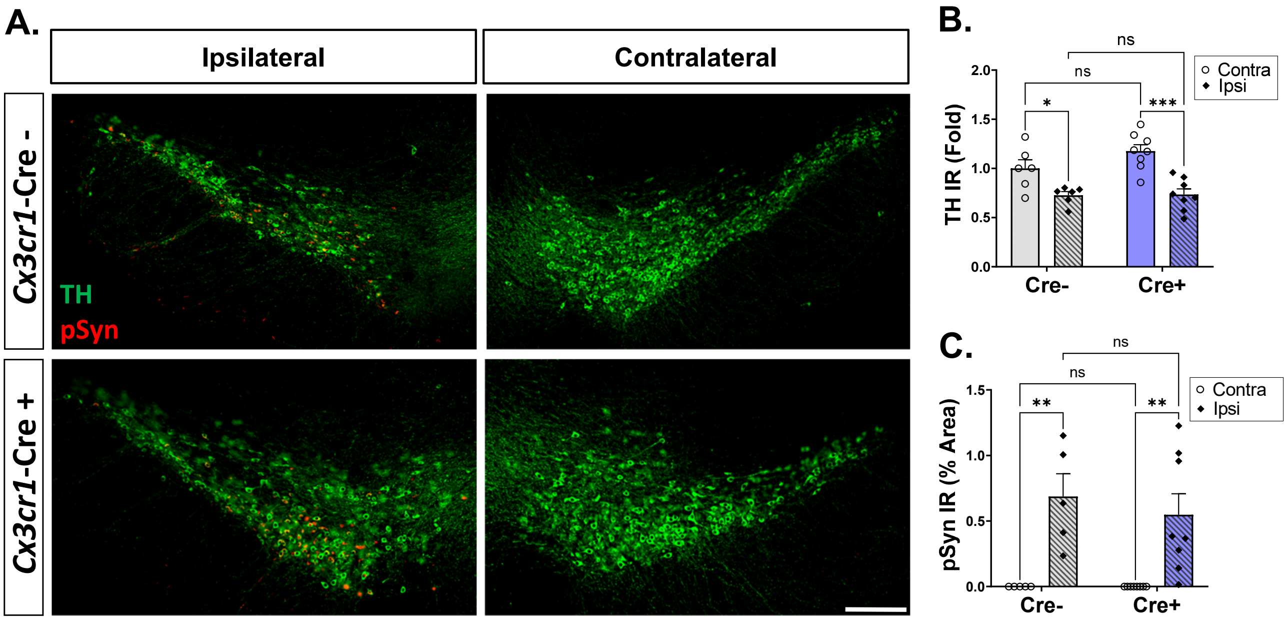

Supplement Figure 6: Microglial *Bmal1* deletion does not impact spontaneous or αSyn PFF-induced dopaminergic neurodegeneration.

A. *Cx3cr1*-Cre^{ERT2};*Bmal1*$^{f/f}$ mice and Cre- littermate controls were treated with tamoxifen at 2mo, then each received a unilateral intrastriatal injection of αSyn PFFs. After 3 months, TH+ neurons and pSyn immunoreactivity were quantified in the SNpc bilaterally. Ipsi: Ipsilateral to PFF injection. Contra: Contralateral to PFF injection.

B. PFF injection caused ipsilateral TH+ neuron loss with no effect of Cre genotype. 2-way ANOVA showed a significant main effect of injection side ($F(1,24)=30.01$, $P<0.0001$), but main effect of Cre genotype ($F(1,24)=2.014$, $P=0.1687$), and interaction ($F(1,24)=1.645$, $P<0.2119$), were not significant.

C. PFF injection caused ipsilateral pSyn accumulation with no effect of Cre genotype 2-way ANOVA showed a significant main effect of injection side ($F(1,22)=25.49$, $P<0.0001$), but main effect of Cre genotype or interaction ($F(1,22)=0.3246$, $P=0.5746$) were not significant.

*P<0.05, **<0.01, ***<0.005 by Tukey’s multiple comparisons test. N=5-8 mice/group, mixed sexes.
